# Disturbance of Mitochondrial Dynamics and Mitochondrial Therapies in Atherosclerosis

**DOI:** 10.3390/life11020165

**Published:** 2021-02-20

**Authors:** Alexander M. Markin, Viktoria A. Khotina, Xenia G. Zabudskaya, Anastasia I. Bogatyreva, Antonina V. Starodubova, Ekaterina Ivanova, Nikita G. Nikiforov, Alexander N. Orekhov

**Affiliations:** 1 Laboratory of Cellular and Molecular Pathology of Cardiovascular System, Institute of Human Morphology, 117418 Moscow, Russia; alexander.markin.34@gmail.com (A.M.M.); nafany905@gmail.com (V.A.K.); nastya.bogatyreva.96@mail.ru (A.I.B.); nikiforov.mipt@googlemail.com (N.G.N.); a.h.opexob@gmail.com (A.N.O.); 2 Laboratory of Angiopathology, Institute of General Pathology and Pathophysiology, 8, Baltiyskaya St., 125315 Moscow, Russia; 3 FSBI National Medical Research Center of Oncology named after N.N. Blokhin of the Ministry of Health of Russia, 115478 Moscow, Russia; ksenya-zabudskaya@mail.ru; 4 Federal Research Centre for Nutrition, Biotechnology and Food Safety, Ustinsky Passage, 109240 Moscow, Russia; avs.ion@yandex.ru; 5 Department of Basic Research, Institute of Atherosclerosis Research, 121609 Moscow, Russia; 6 National Medical Research Center of Cardiology, Institute of Experimental Cardiology, 117418 Moscow, Russia; 7 Institute of Gene Biology, Centre of collective usage, 119344 Moscow, Russia

**Keywords:** atherosclerosis, mitochondrion, mitochondrial dynamics, mtDNA mutation, oxidative stress, inflammation

## Abstract

Mitochondrial dysfunction is associated with a wide range of chronic human disorders, including atherosclerosis and diabetes mellitus. Mitochondria are dynamic organelles that undergo constant turnover in living cells. Through the processes of mitochondrial fission and fusion, a functional population of mitochondria is maintained, that responds to the energy needs of the cell. Damaged or excessive mitochondria are degraded by mitophagy, a specialized type of autophagy. These processes are orchestrated by a number of proteins and genes, and are tightly regulated. When one or several of these processes are affected, it can lead to the accumulation of dysfunctional mitochondria, deficient energy production, increased oxidative stress and cell death—features that are described in many human disorders. While severe mitochondrial dysfunction is known to cause specific and mitochondrial disorders in humans, progressing damage of the mitochondria is also observed in a wide range of other chronic diseases, including cancer and atherosclerosis, and appears to play an important role in disease development. Therefore, correction of mitochondrial dynamics can help in developing new therapies for the treatment of these conditions. In this review, we summarize the recent knowledge on the processes of mitochondrial turnover and the proteins and genes involved in it. We provide a list of known mutations that affect mitochondrial function, and discuss the emerging therapeutic approaches.

## 1. Introduction

Atherosclerosis is a chronic progressive disorder that underlies a large proportion of serious and fatal cardiovascular and neurological events, such as ischemic heart disease, myocardial infarction, and stroke. According to the currently accepted model, the pathogenesis of atherosclerosis is based on both the disturbance of lipid metabolism and chronic inflammation, which directly affects the vascular wall and is present at all stages of disease development. The interplay of these processes includes a wide range of metabolic and signaling cascades, which adds to the disease complexity, but also appears to be promising for identifying novel therapeutic targets. Despite the constant demand for the improvement of diagnostic and therapeutic methods, most of the existing therapies are focused on the reduction in risk factors and alleviation of symptoms. The need for direct anti-atherosclerosis therapies persists. During recent years, personalized medicine appeared in the spotlight as a promising therapeutic approach to treat atherosclerosis, but much work still needs to be done to translate these findings into routine clinical practice [1].

An atherosclerotic lesion is characterized by massive lipid accumulation in the subendothelial space of the arterial wall intima leading to the formation of atherosclerotic plaques. Hypercholesterolemia is still considered as a major cause of atherosclerosis, and normalization of lipid profile helps to slow down the disease’s progression. Especially dangerous are atherogenically modified forms of low-density lipoprotein (LDL), including oxidized LDL, which are not readily metabolized by cells and tend to accumulate [2]. Correspondingly, much research has been performed to better understand the mechanisms and features of hypercholesterolemia and hyperlipidemia in humans and find possible approaches to correcting it [3,4,5]. Although the lipid-lowering approach helps to alleviate the disease, it does not prevent atherosclerosis development at cellular and subcellular level [6]. Understanding these triggers may help in identifying novel treatments that not only slow down the disease, but possibly prevent its development.

Known risk factors of atherosclerosis include both environmental and behavioral factors and genetic predisposition. The importance of the genetic component of atherosclerosis pathogenesis has been relatively well-studied to date. A range of mutations and polymorphisms associated with an increased risk of atherosclerosis have been identified, and the list is constantly growing. Both germline and somatic mutations can be implicated in atherosclerosis. A number of recent studies have clearly demonstrated that mitochondrial DNA (mtDNA) mutations are also associated with atherosclerosis in humans. Along with nuclear, a number of mitochondrial gene mutations are known to cause so-called mitochondrial diseases, in which oxidative phosphorylation is impaired, with often severe consequences for the whole organism [7]. However, beyond the primary mitochondrial diseases, a wide range of chronic human disorders has been shown to be associated with mtDNA mutations and polymorphisms [8]. The development of genetic tools and increasing precision and speed of sequencing methods allowed for establishing a long and ever-growing list of mtDNA mutations associated with human diseases, including atherosclerosis, which may help identifying novel therapeutic targets. For example, to date, researchers from various scientific groups have discovered at least 17 different mtDNA mutations associated with ischemic heart disease, a common complication of atherosclerosis. These changes affect the genes of 6 tRNAs, 12S rRNA subunits, and genes *MT-ND2* and *MT-ND5* for subunits 2 and 5 of the NADH dehydrogenase subunits. Our research team found four mutations in the mitochondrial genome (m.A1555 G in the *MT-RNR1* gene, m.C3256 T in the *MT-TL1* gene, m.G12315A in the *MT-TL2* gene and m.G15059A in the *MT-CYB* gene), which are more often present in lipofibrous plaques compared to the unaffected intima of the aorta. Another mutation discovered by our colleagues, m.G14459A in the *MT-ND6* gene, disrupts the work of the subunit 6 of NADH dehydrogenase, which leads to the appearance of a dysfunctional enzyme. This, in turn, leads to oxidative stress on the mitochondria and damage to the intimal cells themselves [9].

In atherosclerosis, mitochondrial dysfunction is involved in the formation of oxidative stress conditions that facilitate the inflammatory response and lesion development. Moreover, mitochondria are intimately involved in lipid metabolism. In the arterial wall, mitochondrial function is vitally important for proper functioning of all the key cell types involved in atherogenesis: the endothelial cells (ECs), vascular smooth muscular cells (VSMCs), and macrophages that participate in massive lipid accumulation through phagocytosis and maintain the pro-inflammatory milieu in the lesion [10]. Endothelial dysfunction occurs early in the disease development, with reduced nitric oxide (NO) bioavailability, oxidative stress, and increased expression of pro-inflammatory factors and adhesion molecules in the ECs. These changes lead to activation and increase in permeability of the endothelial lining to blood components. As a consequence, the entry of circulating lipoprotein particles into the vascular wall is facilitated. At the same time, the recruitment of leukocytes to the activated cells of the arterial wall promotes the inflammatory response in the newly formed lesion site. Chronic inflammation in the vascular wall associated with atherosclerosis leads to vascular fibroproliferative remodeling and alterations in the extracellular matrix in atherosclerotic plaques [11]. Among the components of mitochondrial dysfunction relevant for atherosclerosis are mitochondria-induced oxidative stress, impaired mitophagy, and metabolic breakdown. Targeting these disturbances appears to be promising for the development of new therapies of atherosclerosis [12,13]. In this review, we will summarize the current knowledge on the input of impaired mitochondrial dynamics in the pathogenesis of atherosclerosis.

## 2. Structure and Functions of Mitochondria and Mitochondrial Genome

Mitochondria are unique semi-autonomous organelles that serve as power stations of eukaryotic cells. According to the theory of endosymbiotic origin, mitochondria derived from α-proteobacteria that were engulfed by a precursor of the modern eukaryotic cell 2.5 billion years ago [14]. A mitochondrion is surrounded by a double membrane, with inner and outer membranes being separated by an intermembrane space and having different composition and functions [15]. Disrupted integrity of the outer membrane or opening the mitochondrial permeability transition pore (PTP) leads to the release of apoptosis-triggering factors (such as cytochrome c) and, ultimately, cell death. The inner membrane hosts the enzymatic machinery responsible for oxidative phosphorylation (OXPHOS), the principal mechanism of aerobic energy production in eukaryotic cells. Its functioning is based on the stepwise movement of electrons from the coenzymes NADH and FADH_2_ to the final electron acceptor O_2_. The OXPHOS system comprises five large multiprotein complexes of the electron transport (respiratory) chain. Complexes I-IV are located in the inner mitochondrial membrane and include several specific electron carriers [16]. The complex V is the ATP synthase that transforms the proton electrochemical gradient to energy stored in ATP [17]. As a by-product of OXPHOS, mitochondria generate reactive oxygen species (ROS) that are, under normal conditions, neutralized by the antioxidant systems and serve mostly as signaling factors. In addition to energy production, mitochondria also act as an important component of cellular Ca^2+^ homeostasis regulators [18]. A substantial part of mitochondrial proteins is encoded by the mitochondria’s own genome, which exists in the form of circular mtDNA. The mtDNA is typically ~16,569 base pairs long and can exist in multiple copies, ranging from 100 to 10,000 per cell. MtDNA contains 37 genes that are responsible for mitochondrial functioning and biogenesis and for aerobic energy production in the cell. There are a wide range of pathologies associated with mutations in “the neglected genome” [19].

Damage of mtDNA is the most common identifiable cause of mitochondrial dysfunction. The proximity of mtDNA to the main sites of ROS production and the lack of protective histones and intrinsically less reliable mechanisms of replication and reparation result in substantially higher rate of mutagenesis as compared to genomic DNA [20]. The replicative apparatus of mtDNA includes polymerase γ, which subunits are encoded by the *POLG1* and *POLG2* genes, Twinkle helicase encoded by the *PEO1* gene, and mtSSBP binding proteins. Mutations in these genes lead to a suppression of replication activity with a subsequent decrease in mtDNA content and increased mutagenesis [21].

The overall effect of mtDNA mutations on mitochondrial function depends on the extent of heteroplasmy (the proportion of affected copies) [22,23]. The degree of heteroplasmy determines both the manifestation and the severity of the disease because a critical number of mutated mtDNA copies is needed for clinical symptoms to become apparent (threshold effect). This pathogenic threshold varies in different tissues according to their metabolism level, explaining why brain and skeletal muscle are relatively often impacted (mitochondrial encephalomyopathies), although potentially every organ and tissue can be affected (multisystem disorders) [24].

Modelling studies have shown that almost 90% of non-proliferating human cells by the age of 70 will have at least 100 mutations per cell, indicating that mtDNA mutations can make a significant contribution to many age-related diseases [25]. Using modern digital methods, it has become possible to quantitatively evaluate and visualize the dynamics of expression of mitochondrial genes and the appearance of their mutations, to identify differences in the regulation of mitochondrial transcriptome and proteome, as well as to identify changes in mitochondrial morphology [26].

## 3. Mechanisms of Mitochondrial Dysfunction

As mentioned above, mitochondrial dysfunction is observed in many human diseases, such as cardiovascular diseases, atherosclerosis, type 2 diabetes, obesity, Parkinson’s disease, Alzheimer’s disease, and other neurodegenerative and autoimmune diseases. Moreover, declining mitochondrial function was shown to contribute to aging processes and the pathogenesis of age-related diseases [27]. In addition, mitochondrial dysfunction can impair the ability of cells to respond to a variety of metabolic challenges, including fatty acid overload [28].

Mitochondrial oxidative stress is known to reduce NO synthesis, enhance the expression of adhesion molecules and secretion of inflammatory cytokines, and mediate oxidation of LDL, which contributes to atherogenesis [29]. Vascular ECs are especially sensitive to oxidative stress. Endothelial dysfunction is characterized by a reduction in NO bioavailability, imbalance between the production of ROS and reactive nitrogen species (RNS), and deficient antioxidant defense [30]. A common term, RONS, that includes both ROS and RNS, is sometimes used to represent the variety of free radicals and molecules of a non-radical nature [31]. The abnormally high RONS levels in aging cells are not readily detected by cellular antioxidant systems, and defective mitochondria escape mitophagy. Excess RONS, along with overloading the mitochondrial matrix with calcium ions, leads to the disruption of transmembrane potential and a decrease in the threshold for PTP opening, which releases RONS, Ca^2+^, nicotinamide adenine dinucleotide (NAD^+^), glutathione and other metabolites into the cytosol, along with apoptosis-triggering factors. This further increases oxidative damage of nuclear DNA, ion channels, transporter proteins and membrane phospholipids [32,33]. Prolonged PTP opening and a high proportion of damaged mitochondria can lead to cell death in the form of mitochondrial permeability transition (MPT)-driven necrosis [34].

The main danger of ROS is that they are able to damage various macromolecules, such as proteins, lipids, and nucleic acids. ROS can cause irreversible DNA damage, such as chain breaks and nucleotide oxidation, as well as DNA modifications. Increased oxidation of free fatty acids contributes to the development of oxidative stress, mitochondrial stress and EPR, and activation of pro-inflammatory signals [35]. Under normal conditions, mitochondrial ROS are neutralized by superoxide dismutase (SOD)1/2. Overexpression of SOD, caused by the stimulation of AMPK kinase activity, helps in reducing the formation of mitochondrial ROS, which prevents damage to the mitochondria and the phenomena caused by the accumulation of fatty acids. Therefore, disruption of these enzymes can increase the oxidative stress in the mitochondria [35,36]. An important component of cellular redox homeostasis is NAD^+^, which was found to be decreased in aging cells. As a result, not only does the pool of redox pairs of NAD^+^/NADH involved in redox reactions decrease, but the activity of NAD^+^-dependent enzymes also decreases [37]. Increasing the cellular NAD^+^ level through its enhanced synthesis or reduced consumption appears to be promising as a therapeutic approach to the treatment of some age-related and oxidative stress-associated conditions. Some of the common drugs with pleiotropic effects, such as statins, were shown to inhibit the prooxidant enzyme NADPH oxidase [38].

Moreover, mitochondria are closely associated with the endoplasmic reticulum (ER) through very dynamic platforms called mitochondria-associated membranes (MAM) that were shown to be necessary for the formation of NLRP3 stimulated by DAMP and pathogen-associated molecular pattern (PAMP) [39]. Free fatty acids contribute to ER stress by releasing Ca^2+^ from the intracellular depots and stimulating the production of ROS. That in turn leads to the activation of the transcription factor NF-κB, which triggers the expression of IRAK2 and secretion of IL-8 and TNFα cytokines. In addition, under the conditions of hyperlipidemia, the NF-κB pathway is also activated in cells expressing TLR2 and TLR4, which leads to the expression of inflammatory and atherogenic genes, and, accordingly, the launch of inflammatory pathways [40].

**Figure 1:**
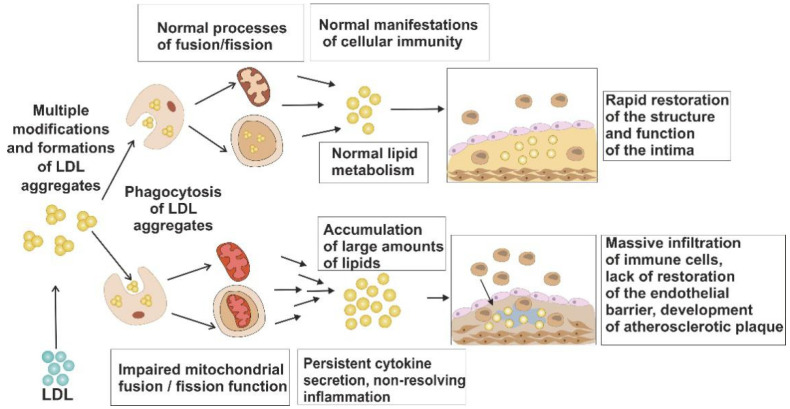
Possible involvement of impaired mitochondrial dynamics in atherosclerosis development. Phagocytic cells, in which normal dynamics is preserved, have a pool of functional mitochondria, which allows them to metabolize the excessive atherogenic lipids (LDL). By contrast, cells with impaired mitochondrial function are prone to lipid accumulation. In the proximity of such cells, atherosclerotic plaque development occurs, accompanied by lipid accumulation and chronic inflammatory response.

The role of mitochondrial dysfunction in atherosclerosis is currently well recognized, although many questions about the details of this involvement remain unanswered, and mitochondria-targeting therapies for atherosclerosis treatment is a topic for future research [28,30]. However, the impact of mitochondrial dysfunction on atherosclerosis hallmark features such as lipid accumulation and chronic inflammation could be distinguished (Figure 1). Our group proposed the following model for this process. Modified LDL entering cells, such as phagocytic macrophages, with intact mitochondrial function, can be metabolized, which prevents massive lipid accumulation and atherosclerotic changes in the arterial wall. By contrast, cells with impaired mitochondrial function, in which the population of healthy mitochondria cannot be restored due to deficient fission and fusion processes, are not able to metabolize the excessive lipids efficiently. That in turn leads to massive lipid accumulation in the arterial wall and atherosclerotic plaque development [13,24].

## 4. Mitochondrial Turnover as Protective Mechanism

**Figure 2:**
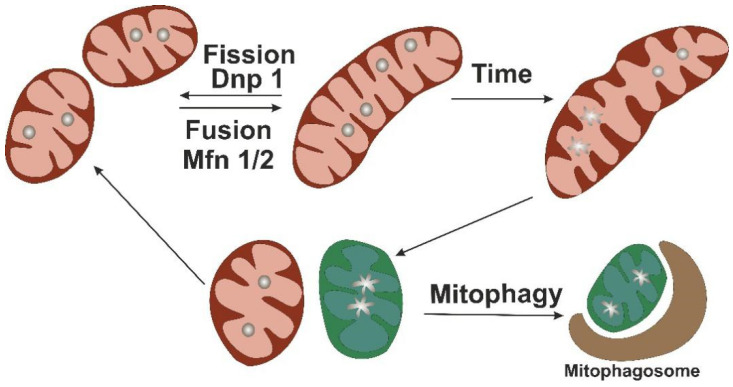
Simplified scheme of mitochondrial turnover. Mitochondrial fission and fusion processes are orchestrated by Dnp 1 and Mfn 1/2, respectively. Accumulation of mtDNA damage that occurs with time may lead to the development of mitochondrial dysfunction. Mitochondrial fission allows for separating the dysfunctional parts of the organelle (shown in green) that are further degraded through mitophagy. The unaffected parts can undergo fusion to form functional organelles.

Mitochondria are dynamic organelles that can adjust their numbers and activity to changing energy needs of the cell and undergo a constant turnover that renews the population of functional organelles and neutralizes the dysfunctional and damaged ones. The key events in mitochondrial turnover are mitochondrial fission, fusion, and mitophagy, a specialized type of autophagy that degrades the excessive or dysfunctional parts of the organelles, separated by fission (Figure 2).

Mitochondrial dynamics ensures the exchange of mtDNA, including damaged and mutated copies, while fission promotes redistribution of mitochondria within the cell and separates dysfunctional mitochondria and damaged mtDNA from the rest of the mitochondrial network. Altered mitochondrial dynamics manifests as the appearance of elongated or malformed organelles or abnormalities of mitochondrial number, and is an early sign of mitochondrial dysfunction that has been described in various human pathologies [41,42,43]. The key molecules of the mitochondrial biogenesis represent promising therapeutic targets for treatment of atherosclerosis. For instance, in atherosclerosis, recovery of mitochondrial dynamics leads to the normalization of the mitochondrial respiration and inhibits stress-induced cell death and necrotic core formation [44].

**Figure 3:**
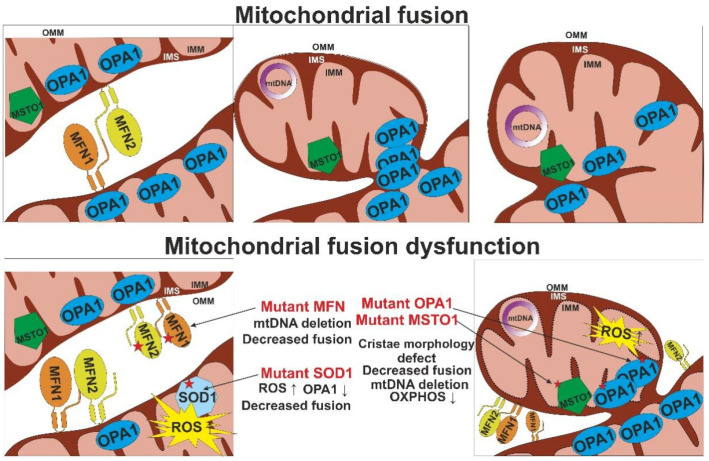
Mitochondrial fusion and its dysfunction. Shown are the main proteins orchestrating the mitochondrial fusion process: mitofusins 1 and 2 (MFN1/2), optic atrophy 1 (OPA1), and misato protein (MSTO1). IMM, inner mitochondrial membrane; IMS, intermembrane space; mtDNA, mitochondrial DNA; OMM, outer mitochondrial membrane; OXPHOS, oxidative phosphorylation; ROS, reactive oxygen species.

The process of mitochondrial fusion is controlled by large GTPases mitofusins 1 and 2 (MFN1 and MFN2) that are located in the outer membrane of the organelle, and optic atrophy 1 (OPA1), located in the inner mitochondrial membrane (Figure 3). In the process of fusion, MFN1 and MFN2 on the surface of the membranes of two mitochondria bind to each other, after which GTP hydrolysis provides for conformational changes necessary for membrane fusion. In the inner mitochondrial membrane, OPA1 is responsible for membrane fusion and the remodeling of mitochondrial cristae and sealing crista junctions [45].

Defective cristae formation is usually observed together with other signs of mitochondrial dynamics dysfunction. In addition to OPA1, the internal structure of mitochondria and the maintenance of crista shape are regulated by the MICOS and F1Fo ATP synthase proteins. Loss of OPA1 leads to cristae defects and mtDNA loss, while deficiency of MIC60 leads to a loss of crista junctions simultaneously with the accumulation of enlarged nucleoids. Autosomal dominant mutations of the *OPA1* gene are associated with disturbances in the processes of oxidative phosphorylation and changes in mitochondrial shape [46].

The fusion process and mitochondrial morphology, as well as chromosome segregation, are regulated by the conserved misato protein, which encodes the *MSTO1* gene in humans. *MSTO1* is expressed in all cells and is localized both in the outer mitochondrial membrane and in the cytoplasm [43].

**Figure 4:**
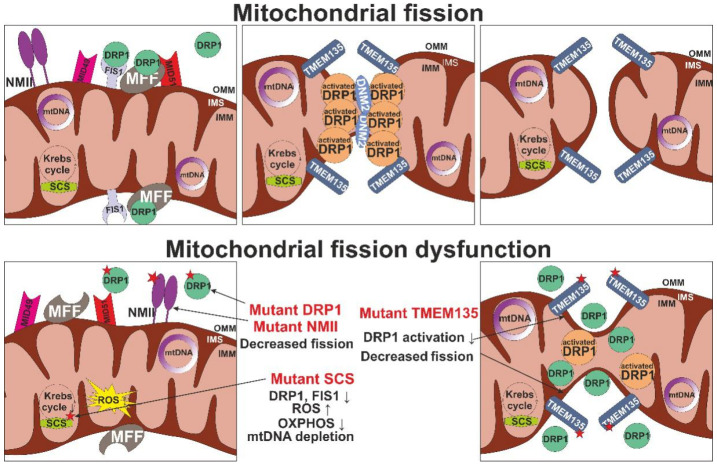
Mitochondrial fission and its dysfunction. Shown are the main proteins involved in mitochondrial fission: non-muscle myosin family II proteins (NMII), mitochondrial fission-1 protein (FIS1), mitochondrial fission factor (MFF), dynamin-1-like protein (DRP1), dynamin 2 (DNM2), mitochondrial dynamics protein 49 and 51 (MID49/51) transmembrane protein 135 (TMEM135). IMM, inner mitochondrial membrane; IMS, intermembrane space; mtDNA, mitochondrial DNA; OMM, outer mitochondrial membrane; OXPHOS, oxidative phosphorylation; ROS, reactive ox-ygen species.

Mitochondrial fission depends on the activity of the cytosolic mitochondrial dynamin-like GTPase DLP1/DRP1 encoded by the *DNM1L* gene, the regulators of which are dynamin 2 (DNM 2), mitochondrial fission factor (MFF), mitochondrial dynamics proteins (MID49, MID51) and mitochondrial fission 1 protein (FIS1) present in the outer mitochondrial membrane (Figure 4). Deficiency of fission leads to the formation of large, elongated mitochondria.

Mitochondrial fission is performed by ATP-dependent molecular motors of non-muscle myosin family II proteins (NMII), represented in the human genome in the form of three isoforms, NMIIA (encoded by *MYH9*), NMIIB (*MYH10*) and NMIIC (*MYH14*), which also take part in recruiting the DRP1 protein to the fission site [47]. Mutations in these genes are known to result in deficient fusion with the formation of elongated organelles, and manifestations of mitochondrial dysfunction through increased oxidative stress and reduced OXPHOS.

The role of autophagy and mitophagy (autophagic degradation of the mitochondria) in maintaining a functional population of the mitochondria in the cell is currently well understood. Disrupted mitophagy leads to the accumulation of damaged mitochondria with increased ROS production. Enhanced expression of p62 nucleoporin protein (an autophagy marker) simultaneously with its impaired degradation in cells with *Atg7* deletion leads to activation of the pathway associated with NRF2 and the antioxidant responsive element (ARE), which contributes to the resistance to oxidative stress. Defective autophagy, including that associated with p62 deficiency with an *Atg7* deletion, disrupts the outflow of cholesterol from cells and can lead to the activation of NLRP3 and a subsequent increase in IL1β secretion [48]. It has been shown that increased mitochondrial fission can lead to accelerated development of atherosclerosis, especially in the presence of diabetes. In endothelial cells, suppression of *Drp1* expression and inhibition of mitochondrial fission due to activation of the AMPK signaling pathway leads to a decrease in mitochondrial ROS production and alleviation of atherosclerotic lesions. Thus, mitochondrial fission promotes atherogenesis by increasing mitochondrial oxidative stress [49].

Palmitic acid (PA) is a saturated fatty acid and, like oxidized LDL, is involved in the development of atherosclerosis. PA induces increased mitochondrial fission in endothelial cells by activating *Drp1* expression, that promotes ROS production and the progression of endothelial dysfunction. Suppression of *Drp1* expression occurs through activation of the Nrf2 pathway and regulation of proteasomes [50]. Recently, *Drp1* was found to be associated with the calcification processes of VSMC and interstitial cells of the heart valve. During osteogenic differentiation, there is a decrease in the mitochondrial membrane potential and increased mitochondrial fission, which can be leveled by the suppression of *Drp1* expression. This may indicate that mitochondrial dynamics plays a role in osteogenic differentiation of atherosclerotic plaque cells [51].

Mitochondrial dynamics drives the adipocyte metabolic change. Induction of thermogenic beige adipocyte is mediated by a dramatic increase in the mitochondria number, while a return to the white fat phenotype is linked to the removal of excessive mitochondria through mitophagy. Hence, mitochondrial turnover can be regarded as a tool to control adipose tissue metabolism and white to beige fat transformation (“beiging”) [52]. Induced beiging of white adipose tissue with an increase in energy expenditure is currently actively studied as a potential therapeutic approach to human diseases associated with impaired metabolism, including diabetes and atherosclerosis. A number of target genes responsible for this process have already been identified, among them cardiolipin synthase (*CRLS1*) and TRG5 receptor, which have been studied in mouse models [53,54]. Impairment of mitochondrial function reduced brown adipocyte-like characteristics of epicardial adipose tissue in mice, leading to vascular inflammation and atherosclerosis [55]. However, pharmacological induction of brown fat failed to prevent atherosclerotic burden in LDLR-deficient mice, despite reducing adiposity and plasma triglyceride level [56]. More studies are needed to explore the therapeutic potential of adipocyte beiging for the treatment of atherosclerosis, diabetes mellitus, and other metabolic diseases [57].

## 5. Mutations of mtDNA Associated with Mitochondrial Dysfunction

Loss-of-function mtDNA mutations can lead to a disruption of mitochondrial morphology and alterations of mitochondrial network through deficiencies in mitochondrial turnover [45]. Table 1 shows the main gene defects that cause different phenotypes of mitochondrial disorders.

mtDNA depletion syndrome represents a genetically and clinically heterogeneous type of mitochondrial disease, which is characterized by an overall reduced amount of mtDNA [68]. Out of the 15 known types of mtDNA depletion syndrome registered in the Online Mendelian Inheritance in Man (OMIM) database, the majority are caused by dysfunction of proteins that are necessary for mtDNA replication, including POLG, C10orf2, MGME1, and TFAM, or for maintaining the mitochondrial dNTP levels, such as TK2, DGUOK, RRM2B, TYMP, SUCLA2, SUCLG1, AGK, MPV17, and SLC25A [69]. However, defects of mitochondrial fission and fusion proteins can also lead to mtDNA depletion. Table 2 describes the most common changes in genes responsible for the processes of mitochondrial dynamics according to the OMIM database [69]. Interestingly, different genetic changes can lead to the same clinical manifestation of the disease. For example, a complex of pathologies related to various types of Charcot–Marie–Tooth disease can be caused by changes in the genes or proteins that control mitochondrial fusion—*Mfn2*, mitochondrial division of *Dnm2* and *Inf2*—as well as proteins that regulate fusion and division processes—Gdap1.

Disruption of fusion processes can lead to the development of severe hereditary pathologies, such as Charcot–Marie–Tooth type 2A disease, hereditary motor and sensory neuropathy VIA, optic nerve atrophy 1, visual atrophy syndrome plus, and Beer’s syndrome. Development of these diseases is associated with the accumulation of multiple mtDNA deletions. Moreover, the heterozygous missense mutation c.A629T (p.D210V) of the *MFN2* gene is also observed. Thus, disruption of the mitochondrial fusion process due to mutations in the *OPA1* and *MFN2* genes can lead to instability and depletion of mtDNA due to new mechanisms that are not fully understood [70]. Disruption of mitochondrial fission was shown to be caused by the c.G2822T mutation in the *MYH14* gene, which leads to the amino acid replacement of R941L in the NMIIC protein sequence [47].

Studies of single-nucleotide variations in the mouse genome revealed a point mutation (T > C) in the splice-donor site adjacent to exon 12 of the *tmem135* gene, which disrupts the functioning of transmembrane protein 135 (TMEM135), which is involved in the regulation of mitochondrial fission and fusion. Expression of the mutant *tmem135* gene leads to an imbalance in the dynamics of mitochondria towards fusion and subsequent increase in size and decrease in the number of mitochondria in cells [71].

Phenotypes similar to those caused by mutations in genes directly responsible for mitochondrial fission and fusion can develop in response to changes in genes that regulate related processes, which are even more common. Mutations in a highly conserved and ubiquitously expressed TDP-43 DNA/RNA-binding protein (TAR DNA-binding protein 43), which is encoded by the *TARDBP* gene, can affect the mitochondrial dynamics indirectly, by regulating the expression of nuclear genes, or directly through regulating the local dynamics. The TDP-43 is predominantly localized in the nucleus, but it is also found in the cytoplasm, and participates in transcription and post-transcriptional RNA modifications. The mutations in the *TARDBP* gene leading to p.Q331K and p.M337V substitutions lead to abnormally increased localization of TDP-43 in the mitochondria and increased mitochondrial fragmentation. In addition, overexpression of non-mutant *TARDBP* leads to impaired mitochondrial movement and alters the expression of DLP1, MFN1, and FIS1 proteins, which suggests that TDP-43 regulates multiple target genes that are involved in the movement and dynamics of mitochondria [41].

Another protein involved in the maintenance of mitochondrial balance is ALEX3, which belongs to the Alex protein family and is encoded by the *ARMCX* gene cluster. In addition, ALEX3 affects mitochondrial transport (trafficking) through interaction with the Kinesin/ Miro1/Trak2 protein complex. ALEX3 can be regulated by the Wnt/PKC signaling cascade, which is involved in the regulation of various mitochondrial functions, including biogenesis, triggering apoptosis, and production of ROS. Noncanonical activation of Wnt/PKC signaling leads to ALEX3 degradation and prevention of defective mitochondrial phenotype development [72]. Miro1, the Rho GTPase of the outer mitochondrial membrane, is encoded by the *RHOT1* gene and plays an important role in the dynamics and transport of the mitochondria, Ca^2+^ homeostasis, and calcium-dependent transition of the mitochondrial form (mitochondrial shape transition (MiST)) and subsequent induction of mitophagy [73]. The heterozygous mutations het c.G815A and het c.C1348T in the *RHOT1* gene have been detected in cells with reduced mitochondrial mass. Both mutations lead to a decrease in the number of contact sites between the mitochondria and the ER, where the Miro1 protein acts as a regulator of Ca^2+^ transporters, resulting in impaired energy production and a subsequent increase in mitophagy.

Furthermore, the combined system PINK1 (*pink-1*) and PRKN (*pdr-1*) recognizes proteins on the outer mitochondrial membrane during cellular damage and mediates the clearance of damaged mitochondria through autophagy and proteasome mechanisms. Mutations of the *pink-1* and *pdr-1* genes lead to a change in the structure and functions of the mitochondria due to the induction of excessive mitochondrial fusion with insufficient mitophagy. In addition, mutations in these genes also increase the proton leakage from the mitochondria, which indicates their ability to participate in other mitochondrial processes. A similar phenomenon was observed in cells bearing mutations in the genes encoding for DRP1 and MFN1 [74]. The presence of such mutations can lead to the persistence of damaged mitochondria, contributing to the development of diseases associated with mitochondrial dysfunction. In addition, PRKN and PINK1 are involved in the degradation of MFN2 through ubiquitination [21]. PINK1 can also phosphorylate and activate Miro1 [75].

Numerous cytosolic enzymes are involved in the regulation of mitochondrial dynamics, including division, fusion, movement, and energy supply. For example, a mutation in the *SOD1* gene, c.G93A, leads to disturbances in the morphology and functional activity of the mitochondria due to a change in the expression of key proteins associated with mitochondrial dynamics. This mutation enhances the expression of DRP1, contributing to the fragmentation and fission of mitochondria, and reduces the expression of OPA1, which leads to the suppression of mitochondrial fusion. However, no change in the activity of the MFN1 and MFN2 proteins is observed. Overexpression of the mutant SOD1G93A is also accompanied by increased apoptotic cell death. It is likely that the accumulation of this mutation in the long term causes mitochondrial toxicity, which may be associated with the development of age-related diseases [42].

Violation of mitochondrial bioenergetics and the subsequent change in the regulation of mitochondrial dynamics can also be caused by lipid overload [75]. In particular, overloading cells with saturated fatty acids can temporarily increase mitochondrial respiration, membrane potential, and ROS production, which in turn mediates the post-translational modification of mitochondrial proteins such as AKAP121, DRP1, and OPA1, leading to increased fragmentation of the mitochondrial network, characterized by a narrow tortuosity tubular morphology. The transcriptional coactivator peroxisome proliferator-activated receptor gamma coactivator 1-alpha (PGC1α) is involved in the regulation of genes implicated in energy metabolism, including proteins associated with mitochondria. Activation of PGC1α can influence the quality control of mitochondria, as well as contribute to the restoration of impaired mitochondrial dynamics, in particular fission [76]. PGC1α activity is regulated by phosphorylation by AMPK kinase and deacetylation due to the activity of the SIRT1 deacetylase enzyme. An increase in PGC1α activity in cells with enhanced mitochondrial fragmentation induces a decrease in the expression level of DRP1 protein, as well as an increase in the expression of MFN1 and LC3II, which is involved in the regulation of autophagy. Thus, PGC1α enhances mitochondrial fusion and biogenesis.

To date, many genes have been identified that are indispensable for maintaining mtDNA stability, including *POLG*, *POLG2*, and *PEO1*, encoding key proteins of the mechanism of replication of the main mtDNA, *DNA2* and *MGME1*, encoding proteins involved in the repair and maintenance of mtDNA, *TP*, *TK2*, *DGUOK*, *SLC25A4*, *RRM2B*, *SUCLA2*, *SUCLG1*, and *ABAT*, encoding proteins that preserve the mitochondrial nucleotide pool, and *OPA1*, *MFN2*, and *FBXL4*, encoding proteins involved in mitochondrial dynamics and remodeling of mitochondrial membranes. Mutations in these genes lead to impaired functional activity and mitochondrial morphology, which can lead to the development of many age-related diseases [77].

Disturbances in the functioning of the tricarboxylic acid cycle also lead to disruption of the processes of mitochondrial dynamics. Thus, one of the most important enzymes involved in the tricarboxylic acid cycle is succinyl coenzyme A synthetase (succinyl coenzyme A synthetase, SCS). It is an α-heterodimer consisting of two subunits–α (SCS-α), encoded by the *SUCLG1* gene, and β (SCS β), which in turn has two isoforms, known as the ADP-forming subunit (SCS A-β), encoded by *SUCLA2*, and the GDP-forming β-subunit (SCS G-β), encoded by *SUCLG2*. Mutations in the *SUCLA2* gene impair SCS A-β function, which leads to mitochondrial dysfunction, impaired oxidative phosphorylation and increased ROS production, as well as mtDNA depletion [78]. The latter phenomenon is associated with the inactivation of mitochondrial nucleoside diphosphate kinase (NDPK), leading to the impaired metabolism of mitochondrial deoxyribonucleotide (dNTP), as well as with a decrease in the expression of proteins associated with mtDNA replication, such as Twinkle and POLG. In addition, the loss of SCS A-β causes a significant decrease in the expression level of both mitochondrial fusion proteins Mfn2 and OPA1, and Fis1 and DLP1 fission proteins. At the same time, an increase in the mitochondrial size was described, which may be due to the fact that SCS is the main source of GTP in the mitochondria, and inactivation of the enzyme leads to a change in the production of mitochondrial GTP. That can have a significant effect on the operation of OPA1, despite the fact that the level of its expression in cells is reduced. In this regard, the balance of mitochondrial fusion and fission changes.

Interestingly, disruption of the peroxisome assembly process results in a phenotype similar to that caused by dysfunctional mitochondrial fission. In fact, peroxisomes participate in mitochondrial fission. Inactivation of the Pex16 protein, which is located in the ER and is one of the key factors in the assembly of the peroxisomal membrane and the import of peroxisome membrane proteins, blocks mitochondrial fission, which leads to the formation of the phenotype described previously [79].

An imbalance in the functioning of genes for mitochondrial dynamics can disrupt the work of more functioning systems. For example, two missense mutations in the *MSTO1* gene (c.C1033T (p.R345C) and c.C1128A; (p.F376L)), as well as two heterozygous mutations (c.C971T (p.T324I) and c.G966A), lead to increased mitochondrial fragmentation due to impaired fusion and transport of mitochondria [43]. In addition, the c.G22 A mutation (p.V8M) was detected, which causes a decrease in the MSTO1 protein content, as well as mitochondrial fragmentation, aggregation, decreased mitochondrial network continuity, and decreased fusion activity [80].

A large part of mitochondrial proteins is encoded by nuclear DNA, and the normal functioning of mitochondria depends, among other things, on the entry of nuclear gene products into the mitochondria. Thus, protein precursors (pre-proteins) are recognized and imported into the mitochondria using the translocase of outer mitochondrial membrane (Tom) complex. Correspondingly, defects in this transport complex can lead to profound mitochondrial dysfunction, including impaired dynamics. The Tom complex consists of seven components: Tom40, central channel protein, receptor proteins Tom20, Tom22 and Tom70, as well as Tom5, Tom6 and Tom7, which regulate the stability of the complex. The Tom20 and Tom70 proteins recognize the precursor proteins and transfer them to the Tom22 receptor and on to the Tom40 import channel. In addition, Tom22 is also involved in the assembly of the Tom complex [81]. Under hyperglycemic conditions, Tom22 regulates mitochondrial fusion and fission through direct binding to the Mfn1 gene. Knockdown Tom22 reduces the expression of the Mfn1 gene and increases the expression of Fis1, Mff and Drp1, which leads to a shift in the balance of mitochondrial dynamics towards fission [81].

## 6. Directions for Mitochondrial Therapy Development

Developing effective treatments for mitochondrial diseases has proven to be an extremely challenging task. For various congenital metabolic disorders, therapeutic approaches include reducing metabolic load through dietary changes, removing toxic metabolites, enzyme therapy, or organ (liver or bone marrow) transplantation. However, the situation with mitochondrial disorders is somewhat more complicated due to the extreme genetic and phenotypic heterogeneity of mitochondrial disorders, which makes it very difficult to collect sufficiently large groups of patients to conduct adequately powerful, randomized, double-blind, placebo-controlled clinical trials. A persistent problem in clinical trials of mitochondrial diseases is the lack of clinically significant, universally agreed upon and confirmed criteria for evaluating the results [82]. However, some of the pathologies could be corrected, at least to some extent. These include defects in CoQ10 biosynthesis, which can manifest as infant-onset encephalomyopathy, nephrotic syndrome, ataxia, seizures, or isolated myopathy [83]. Early initiation of CoQ10 supplementation helped in achieving positive clinical outcomes [84]. However, it should be noted that not all patients respond to CoQ10 therapy [85,86].

Diagnostic approaches to mitochondrial diseases are also challenging. Genetic methods include determining the number of copies of mtDNA in cells or assessing the content of specific microRNAs (miRNAs). The first, the amount (copy number) of mtDNA in peripheral blood cells (mainly leukocytes and platelets), can serve as an indicator of the mitochondrial function. The analysis of microRNAs, which are small noncoding RNAs that regulate gene expression, can help in assessing mitochondrial functional activity. Mitochondrial miRNAs (mitomiR) have been identified and shown to be involved in post-transcriptional regulation of mitochondrial gene expression (miR-1, miR-210, miR-338) and the metabolism, in particular OXPHOS (miR-696, miR-532, miR-690, miR-345-3p), ROS production (miR-762, miR-181c) and lipid metabolism. Many mitomiRs take part both in the regulation of mitochondrial dynamics. For instance, miR-484 inhibits the expression of FIS1, miR-210 and miR-30 - DRP1. Some mitomiRs were shown to be involved in the activation of apoptosis processes. In particular, miR-146a, miR-34a, and miR-181a suppress the antioxidant and antiapoptotic mitochondrial protein expression of Bcl-2, which leads to an increase in ROS production, PTP opening, and the activation of caspase-1 and 3. Thus, the activity of some mitomiRs can contribute to mitochondrial dysfunction, increased oxidative stress, chronic inflammation, and increased cell death [18]. At present, most patients with mitochondrial diseases are offered symptomatic treatment, while the search for novel and effective therapies continues [86].

Therapeutic approaches that are designed to correct the mitochondrial balance appear to be promising. Changes in the mitochondrial morphology, impaired mitochondrial bioenergetics, an increase in mitochondrial lipid peroxides, a decrease in ATP, and mitochondrial dysfunction further increase the risk of metabolic complications. Mitochondrial outer membrane protein mitoNEET (also known as CDGSH iron sulfur domain 1 protein) can affect mitochondrial activity. MitoNEET is a redox enzyme that may promote the oxidation of NADH to facilitate enhanced glycolysis in the cytosol [87]. Changes in mitoNEET expression can modulate electron transfer activity by dysregulating ROS concentrations and mitochondrial iron transport into the matrix [29].

Stimulation of mitochondrial biogenesis in order to compensate for dysfunctional organelles bears a risk that the proliferation of damaged mitochondria also increases, negatively affecting the therapy’s efficacy. Mitochondrial biogenesis is under complex regulatory control, requiring coordinated transcription of multiple proteins encoded in two cellular compartments. This allows for in vivo adaptation of mitochondrial function depending on the availability of nutrients and oxygen, hormonal signals, various metabolic requirements, and the rate of cell proliferation. Transcription coactivator PPAR-γ coactivator 1-α (PGC-1α) coordinates mitochondrial biogenesis through a cascade of nuclear-encoded hormone receptors, transcription factors and transcription coactivators, including PPARs, estrogen-related receptors, thyroid hormone receptors, nuclear respiratory factors NRF1 and 2, and transcription factors CREB and YY1 [88]. Another prospective therapeutic approach is focused on mitochondrial dynamics and mitophagy [89,90]. Genetic causes of mitochondrial dynamics abnormalities include mutations in *MFN2* or *OPA1* genes, which manifest as Charcot–Marie–Tooth type 2A and autosomal dominant optic nerve atrophy, respectively [91,92], as well as a violation of mitochondrial division caused by mutations affecting Drp1 and Mff [93]. Currently, research is being carried out on specific inhibitors of mitochondrial fusion (M-hydrazone) and fission (MDIVI-1 and P110) [94,95,96].

The search for potential therapeutic drugs among non-traditional drugs also continues. For example, a substance isolated from sunphenon epigallocatechin gallate (EGCg) green tea extract has been tested in clinical trials. This work showed the presence of an antioxidant, antiapoptotic effect. In addition, sunphenon EGCg is described as a modulator of cell survival and mitochondrial function [97]. Most of the investigated drugs aimed at modulating mitochondrial function are antioxidant drugs (R + pramipexole, EGCg, DL-3-n-butylphthalide (NBP), mitoQ, resveratrol), while one was described as a molecule that enhances mitochondrial function (S-equol), and one substance was positioned as an antiapoptotic agent (Dimebon) [98].

The accumulating information on the different genomic and mtDNA mutations and their input in the disease pathogenesis can contribute significantly to the development of personalized medicine. Patients with different predominating pathogenetic aspects (e.g., inflammatory or metabolic) may benefit more from tailored medicines and enjoy improved safety of applied treatments. The detection of mutations with high clinical significance and accurate genetic counseling in individuals with mitochondrial atherosclerosis can inform treatment strategies [99].

The possibility of correcting the mitochondrial dysfunction in a selective manner by using mitochondria-targeting antioxidants or removing dysfunctional organelles by phototheranostic approaches opens up new and exciting opportunities for atherosclerosis treatment. Such approaches are already being used in the field of cancer therapy. An important line of future research appears to be translating this knowledge to atherosclerosis treatment as well as developing novel approaches that can selectively target the mitochondria of arterial wall cells.

## 7. Conclusions

The role of mitochondrial dysfunction in atherosclerosis is currently well recognized. Of special interest is the possible explanation that the local appearance of mtDNA mutations and distribution of cells with manifest mitochondrial dysfunction may provide for the focal formation of atherosclerotic lesions in the arterial wall. Appearance of mtDNA mutations is one of the major causes of mitochondrial dysfunction that can be detected and analyzed. As a matter of fact, some mtDNA mutations are already being explored as possible diagnostic markers. Future studies will help in expanding and refining the list of mtDNA mutations and variants relevant to atherosclerosis. Among the emerging drugs for treatment of diseases associated with mitochondrial dysfunction are several molecules that may help in correcting mitochondrial balance through influencing mitochondrial fission and fusion processes. Some of them have shown interesting effects in preclinical and clinical studies. However, more studies are needed to create mitochondria-targeting therapies suitable for widespread use.

## Figures and Tables

**Table 1:** Phenotypes associated with disrupted mitochondrial turnover.

Gene	Gene Function	Cellular Phenotype	Cristae Abnormalities	Mitochondrial DNA/Nucleoid Aberrancy	Ref.
*MFN1* *MFN2* *MFN1*+*MFN2*	OMM fusion; MFN2 key regulator of the mitochondria-ER contact sites tethering; MFN1; involved in the OPA1-dependent IMM fusion	Reduced cell growth and oxygen consumption, loss of transmembrane potential	Sparse cristae with swollen, fragmented mitochondria with reduced COX activity	Loss of mtDNA (DKO MFN1 and MFN2) and loss of nucleotide (КО, DKO)	[58,59]
*OPA1*	IMM fusion	Loss of transmembrane potential (mammalian cell lines); reduced body weight, muscle atrophy and weakness, kyphosis and hair greying (inducible conditional deletion in skeletal muscle)	Crystae p-depletion, dilation, disorganization	mtDNA loss	[60,61,62]
*MSTO1*	cytosolic mitochondrial fusion regulator	Fragmented mitochondrial network, Enlarged lysosomes	-	Nucleoid clumping segregation of mtDNA; reduced mtDNA copy number	[63]
*RP1*	OMM fission	Delayed apoptosis, reduced membrane potential and ATP synthesis	Densly packed cristae (mito-bulbs)	Nucleoid clustering	[64,65]
*MFF*	OMM fission	Premature death, cardiomyopathy	Vacuolated mitochondria with malformed cristae	mtDNA levels decline	[66]
*MIC60*	CJs formation and assembly	Impaired mitochondrial dynamics	Loss of CJs leading to cristae separated from the IBM	Increased nucleoids, decreased transcription of mtDNA-encoded genes	[67]

MFN1 & 2: mitofusins 1 and 2; OPA1: optic atrophy type 1, DRP-1: dynamin-related protein 1; MFF: mitochondrial fission factor; *MIC60*: core component of the MICOS complex; OMM: outer mitochondrial membrane; IMM: inner mitochondrial membrane; IBM: inner boundary membrane; CJs: Crista junctions.

**Table 2:** Aberrant mitochondrial dynamics in the human pathology.

Gene	OMIM	Phenotype	Inheritance
*Fusion genes*			
*MFN2*	608507	Charcot–Marie–Tooth disease type 2A	AD/AR
		Hereditary motor and sensory neuropathy VIA	AD
*ОРА1*	605290	Optic atrophy 1	AD
		Optic atrophy plus syndrome	AD
		Behr syndrome	AR
*Fission genes*			
*DNM1L*	603850	Encephalopathy	AR/AD
		Optic atrophy 5	AD
*MFF*	614785	Encephalopathy	AR
*MIEF2*	615498	Mitochondrial myopathy	AR
*DNM2*	602378	Centronuclear myopathy 1	AD
		Charcot–Marie–Tooth disease, axonal type 2M	AD
		Charcot–Marie–Tooth disease, dominant intermediate B	AD
		Lethal congenital contracture syndrome 5	AR
*INF2*	610982	Charcot–Marie–Tooth disease type E	AD
		Focal segmental glomerulosclerosis	AD
*genes-regulators of mitochondrial dynamics*			
*MSTO1*	617619	Myopathy and ataxia	AR/AD
*SLC25A46*	610826	Pontocerebellar hypoplasia type 1	AR
		Hereditary sensory motor neuropathy	AR
		Optic atrophy spectrum disorders	AR
*GDAP1*	606598	Charcot–Marie–Tooth disease type 4A	AR
		Charcot–Marie–Tooth disease type 2K	AR/AD
		Charcot–Marie–Tooth disease type A	AR
		Charcot–Marie–Tooth disease with vocal cord paresis	AR

AD, autosomal dominant; AR, autosomal recessive; OMIM, Online Mendelian Inheritance in Man^®;^ *MFN2*, mitofusins 2; *OPA1*, optic atrophy type 1; *MSTO1*, misato homolog 1; *DNM1L*, dynamin 1 like gene; *MFF*, mitochondrial fission factor; *MIEF2*, gene coding mitochondrial dynamics protein MID49; *DNM2*, dynamin 2; *SLC25A46*, solute carrier family 25 member 46; *GDAP1*, ganglioside-induced differentiation associated protein 1; *INF2*, inverted formin 2.

## References

[1] Peterlin A., Petrovič D., Peterlin B. (2019). Screening for Rare Genetic Variants Associated with Atherosclerosis: Opportunity for Personalized Medicine. Curr. Vasc. Pharmacol..

[2] Sukhorukov V.N., Karagodin V.P., Orekhov A.N. (2016). Atherogenic modification of low-density lipoproteins. Biomed. Khim..

[3] Steinberg D. (2002). Atherogenesis in perspective: Hypercholesterolemia and inflammation as partners in crime. Nat. Med..

[4] Friedman A., Hao W. (2015). A mathematical model of atherosclerosis with reverse cholesterol transport and associated risk factors. Bull. Math. Biol..

[5] Formanowicz D., Krawczyk J.B., Perek B., Formanowicz P. (2019). A Control-Theoretic Model of Atherosclerosis. Int. J. Mol. Sci..

[6] Mushenkova N.V., Summerhill V.I., Silaeva Y.Y., Deykin A.V., Orekhov A.N. (2019). Modelling of atherosclerosis in genetically modified animals. Am. J. Transl. Res..

[7] Gorman G.S., Chinnery P.F., DiMauro S., Hirano M., Koga Y., McFarland R., Suomalinen A., Thorburn D.R., Zeviani M., Turnbull D.M. (2016). Mitochondrial diseases. Nat. Rev. Dis Primers.

[8] Carelli V., La Morgia C. (2018). Clinical syndromes associated with mtDNA mutations: Where we stand after 30 years. Essays Biochem..

[9] Poznyak A.V., Ivanova E.A., Sobenin I.A., Yet S.F., Orekhov A.N. (2020). The Role of Mitochondria in Cardiovascular Diseases. Biology.

[10] Weber C., Noels H. (2011). Atherosclerosis: Current pathogenesis and therapeutic options. Nat. Med..

[11] Bajraktari A., Bytyçi I., Henein M.Y. (2020). The Relationship between Coronary Artery Wall Shear Strain and Plaque Morphology: A Systematic Review and Meta-Analysis. Diagnostics.

[12] Orekhov A.N., Nikiforov N.N., Ivanova E.A., Sobenin I.A. (2020). Possible Role of Mitochondrial DNA Mutations in Chronification of Inflammation: Focus on Atherosclerosis. J. Clin. Med..

[13] Orekhov A.N., Poznyak A.V., Sobenin I.A., Nikifirov N.N., Ivanova E.A. (2019). Mitochondrion as a selective target for treatment of atherosclerosis: Role of mitochondrial DNA mutations and defective mitophagy in the pathogenesis of atherosclerosis and chronic inflammation. Curr. Neuropharmacol..

[14] Wallace D.C. (2018). Mitochondrial genetic medicine. Nat. Genet..

[15] McStay G.P. (2017). Complex formation and turnover of mitochondrial transporters and ion channels. J. Bioenerg. Biomembr..

[16] Wilson D.F. (2017). Oxidative phosphorylation: Regulation and role in cellular and tissue metabolism. J. Physiol..

[17] Nesci S., Pagliarani A. (2019). Emerging Roles for the Mitochondrial ATP Synthase Supercomplexes. Trends Biochem. Sci..

[18] Song R., Hu X., Zhang L. (2019). Mitochondrial MiRNA in Cardiovascular Function and Disease. Cells.

[19] Pesole G., Allen J.F., Lane N., Martin W., Rand D.M., Schatz G., Saccone C. (2012). The neglected genome. EMBO Rep..

[20] Stein A., Sia E.A. (2017). Mitochondrial DNA repair and damage tolerance. Front. Biosci..

[21] Ahmed N., Ronchi D., Comi G.P. (2015). Genes and pathways involved in adult onset disorders featuring muscle mitochondrial DNA instability. Int. J. Mol. Sci..

[22] Sobenin I.A., Mitrofanov K.Y., Zhelankin A.V., Sazonova M.A., Postnov A.Y., Revin V.V., Bobryshev Y.V., Orekhov A.N. (2014). Quantitative assessment of heteroplasmy of mitochondrial genome: Perspectives in diagnostics and methodological pitfalls. BioMed Res. Int..

[23] Wallace D.C., Chalkia D. (2013). Mitochondrial DNA genetics and the heteroplasmy conundrum in evolution and disease. Cold Spring Harb. Perspect. Biol..

[24] Glanz V.Y., Sobenin I.A., Grechko A.V., Yet S.F., Orekhov A.N. (2020). The role of mitochondria in cardiovascular diseases related to atherosclerosis. Front. Biosci..

[25] Li H., Slone J., Fei L., Huang T. (2019). Mitochondrial DNA Variants and Common Diseases: A Mathematical Model for the Diversity of Age-Related mtDNA Mutations. Cells.

[26] Yim A., Koti P., Bonnard A., Marchiano F., Urrbaum M.D., Garcia-Perez C., Villaveces J., Gamal S., Cardone G., Perocchi F. (2020). mitoXplorer, a visual data mining platform to systematically analyze and visualize mitochondrial expression dynamics and mutations. Nucleic Acids Res..

[27] Moro L. (2019). Mitochondrial Dysfunction in Aging and Cancer. J. Clin. Med..

[28] Tin A., Grams M.E., Ashar F.N., Lane J.A., Rosenberg A.Z., Grove M.L., Boerwinkle E., Selvin E., Coresh J., Pankratz N. (2016). Association between mitochondrial DNA copy number in peripheral blood and incident CKD in the atherosclerosis risk in communities study. J. Am. Soc. Nephrol..

[29] Hernández-Aguilera A., Rull A., Rodríguez-Gallego E., Riera-Borrull M., Luciano-Mateo F., Camps J., Menéndez J.A., Joven J. (2013). Mitochondrial Dysfunction: A Basic Mechanism in Inflammation-Related Non-Communicable Diseases and Therapeutic Opportunities. Mediat. Inflamm..

[30] Förstermann U., Xia N., Li H. (2017). Roles of Vascular Oxidative Stress and Nitric Oxide in the Pathogenesis of Atherosclerosis. Circ. Res..

[31] Di Meo S., Reed T.T., Venditti P., Victor V.M. (2016). Role of ROS and RNS Sources in Physiological and Pathological Conditions. Oxidative Med. Cell Longev..

[32] Briston T., Roberts M., Lewis S., Powney B., Staddon J.M., Szabadkai G., Duchen M.R. (2017). Mitochondrial permeability transition pore: Sensitivity to opening and mechanistic dependence on substrate availability. Sci. Rep..

[33] Rottenberg H., Hoek J.B. (2017). The path from mitochondrial ROS to aging runs through the mitochondrial permeability transition pore. Aging Cell.

[34] Galluzzi L., Vitale I., Aaronson S.A., Abrams J.M., Adam D., Agostinis P., Alnemri E.S., Altucci L., Amelio I., Andrews D.W. (2018). Molecular mechanisms of cell death: Recommendations of the Nomenclature Committee on Cell Death 2018. Cell Death Differ..

[35] Kaludercic N., Di Lisa F. (2020). Mitochondrial ROS Formation in the Pathogenesis of Diabetic Cardiomyopathy. Front. Cardiovasc. Med..

[36] Eirin A., Lerman A., Lerman L.O. (2014). Mitochondrial injury and dysfunction in hypertension-induced cardiac damage. Eur. Heart J..

[37] Yoshino J., Baur J.A., Imai S.I. (2018). NAD(+) Intermediates: The Biology and Therapeutic Potential of NMN and NR. Cell Metab..

[38] Moon G.J., Kim S.J., Cho Y.H., Ryoo S., Bang O.Y. (2014). Antioxidant effects of statins in patients with atherosclerotic cerebrovascular disease. J. Clin. Neurol..

[39] Missiroli S., Patergnani S., Caroccia N., Pedriali G., Perrone M., Previati M., Wieckowski M.R., Giorgi C. (2018). Mitochondria-associated membranes (MAMs) and inflammation. Cell Death Dis..

[40] Shah P.K., Lecis D. (2019). Inflammation in atherosclerotic cardiovascular disease. F1000Research.

[41] Wang W., Li L., Lin W.L., Dickson D.W., Petrucelli L., Zhang T., Wang X. (2013). The ALS disease-associated mutant TDP-43 impairs mitochondrial dynamics and function in motor neurons. Hum. Mol. Genet..

[42] Wang H., Yi J., Li X., Xiao Y., Dhakal K., Zhou J. (2018). ALS-associated mutation SOD1G93A leads to abnormal mitochondrial dynamics in osteocytes. Bone.

[43] Nasca A., Scotton C., Zaharieva I., Neri M., Selvatici R., Magnusson O.T., Gal A., Weaver D., Rossi R., Armaroli A. (2017). Recessive mutations in MSTO1 cause mitochondrial dynamics impairment, leading to myopathy and ataxia. Hum. Mutat..

[44] Forrester S.J., Griendling K.K. (2017). Mitochondrial Respiration and Atherosclerosis: R-E-S-P-I-R-E. Find Out What it Means to Mϕ (and VSMC). Arter. Thromb. Vasc. Biol..

[45] Kondadi A.K., Anand R., Reichert A.S. (2019). Functional Interplay between Cristae Biogenesis, Mitochondrial Dynamics and Mitochondrial DNA Integrity. Int. J. Mol. Sci..

[46] Viscomi C., Zeviani M. (2017). MtDNA-maintenance defects: Syndromes and genes. J. Inherit. Metab. Dis..

[47] Almutawa W., Smith C., Sabouny R., Smit R.B., Zhao T., Wong R., Lee-Glover L., Desrochers-Goyette J., Ilamathy H.S., Care4Rare Canada Consortium (2019). The R941L mutation in MYH14 disrupts mitochondrial fission and associates with peripheral neuropathy. EBioMedicine.

[48] Grootaert M.O.J., Roth L., Schrijvers D.M., De Meyer G.R.Y., Martinet W. (2018). Defective Autophagy in Atherosclerosis: To Die or to Senesce?. Oxidative Med. Cell Longev..

[49] Wang Q., Zhang M., Torres G., Wu S., Ouyang C., Xie Z., Zou M.H. (2017). Metformin suppresses diabetes-accelerated atherosclerosis via the inhibition of Drp1-mediated mitochondrial fission. Diabetes.

[50] Zhu Y., Li M., Lu Y., Li J., Ke Y., Yang J. (2019). Ilexgenin A inhibits mitochondrial fiss, ion and promote Drp1 degradation by Nrf2-induced PSMB5 in endothelial cells. Drug Dev. Res..

[51] Rogers M.A., Maldonado N., Hutcheson J.D., Goettsch C., Goto S., Yamada I., Faits T., Sesaki H., Aikawa M., Aikawa E. (2017). Dynamin-Related Protein 1 Inhibition Attenuates Cardiovascular Calcification in the Presence of Oxidative Stress. Circ. Res..

[52] Lu X. (2019). Maintaining mitochondria in beige adipose tissue. Adipocyte.

[53] Sustarsic E.G., Ma T., Lynes M.D., Larsen M., Karavaeva I., Havelund J.F., Nielsen C.H., Jedrychowski M.P., Moreno-Torres M., Lundh M. (2018). Cardiolipid synthesis in brown and beige fat mitochondria is essential for systemic energy homeostasis. Cell Metab..

[54] Velazquez-Villegas L.A., Perino A., Lemos V., Zietak M., Nomura M., Pols T.W.H., Schoonjans K. (2018). TGR5 signalling promotes mitochondrial fission and beige remodelling of white adipose tissue. Nat. Commun..

[55] Tang Y., He Y., Li C., Mu W., Zou Y., Liu C., Qian S., Zhang F., Pan J., Wang Y. (2018). RPS3A positively regulates the mitochondrial function of human periaortic adipose tissue and is associated with coronary artery diseases. Cell Discov..

[56] Worthmann A., Schlein C., Berbée J.F.P., Rensen P.C.N., Heeren J., Bartelt A. (2019). Effects of Pharmacological Thermogenic Adipocyte Activation on Metabolism and Atherosclerotic Plaque Regression. Nutrients.

[57] Lizcano F. (2019). The Beige Adipocyte as a Therapy for Metabolic Diseases. Int. J. Mol. Sci..

[58] Chen H., McCaffery J.M., Chan D.C. (2007). Mitochondrial fusion protects against neurodegeneration in the cerebellum. Cell.

[59] Silva Ramos E., Motori E., Bruser C., Kuhl I., Yeroslaviz A., Ruzzenente B., Kauppila J.H.K., Busch J.D., Hultenby K., Habermann B.H. (2019). Mitochondrial fusion is required for regulation of mitochondrial DNA replication. PLoS Genet..

[60] Olichon A., Baricault L., Gas N., Guillou E., Valette A., Belenguer P., Lenaers G. (2003). Loss of OPA1 perturbates the mitochondrial inner membrane structure and integrity, leading to cytochrome c release and apoptosis. J. Biol. Chem..

[61] Chen L., Liu T., Tran A., Lu X., Tomilov A.A., Davies V., Cortopassi G., Chiamvimonvat N., Bers D.M., Votruba M. (2012). OPA1 mutation and late-onset cardiomyopathy: Mitochondrial dysfunction and mtDNA instability. J. Am. Heart Assoc..

[62] Tezze C., Romanello V., Desbats M.A., Fadini G.P., Albeiro M., Favaro G., Ciciliot S., Soriano M.E., Morbidoni V., Cerqua C. (2017). Age-Associated Loss of OPA1 in Muscle Impacts Muscle Mass, Metabolic Homeostasis, Systemic Inflammation, and Epithelial Senescence. Cell Metab..

[63] Donkervoort S., Sabouny R., Yun P., Gauquelin L., Chao K.R., Hu Y., Al Khatib I., Topf A., Mohassel P., Cummings B.B. (2019). MSTO1 mutations cause mtDNA depletion, manifesting as muscular dystrophy with cerebellar involvement. Acta Neuropathol..

[64] Ban-Ishihara R., Ishihara T., Sasaki N., Mihara K., Ishihara N. (2013). Dynamics of nucleoid structure regulated by mitochondrial fission contributes to cristae reformation and release of cytochrome c. Proc. Natl. Acad. Sci. USA.

[65] Ishihara T., Ban-Ishihara R., Maeda M., Matsunaga Y., Ichimura A., Kyogoku S., Aoki H., Katada S., Nakada K., Nomura M. (2015). Dynamics of mitochondrial DNA nucleoids regulated by mitochondrial fission is essential for maintenance of homogeneously active mitochondria during neonatal heart development. Mol. Cell Biol..

[66] Chen H., Ren S., Clish C., Jain M., Mootha V., McCaffery J.M., Chan D.C. (2015). Titration of mitochondrial fusion rescues Mff-deficient cardiomyopathy. J. Cell Biol..

[67] Li H., Ruan Y., Zhang K., Jian F., Hu C., Miao L., Gong L., Sun L., Zhang X., Chen S. (2016). Mic60/Mitofilin determines MICOS assembly essential for mitochondrial dynamics and mtDNA nucleoid organization. Cell Death Differ..

[68] El-Hattab A.W., Scaglia F. (2013). Mitochondrial DNA depletion syndromes: Review and updates of genetic basis, manifestations, and therapeutic options. Neurother J. Am. Soc. Exp. Neuro Ther..

[69] Amberger J.S., Bocchini C.A., Schiettecatte F., Scott A.F., Hamosh A. (2015). OMIM.org: Online Mendelian Inheritance in Man (OMIM®), an online catalog of human genes and genetic disorders. Nucleic Acids Res..

[70] Chandhok G., Lazarou M., Neumann B. (2018). Structure, function, and regulation of mitofusin-2 in health and disease. Biol. Rev. Camb. Philos. Soc..

[71] Lee W.H., Higuchi H., Ikeda S., Macke E.L., Takimoto T., Pattnaik B.R., Liu C., Chu L.F., Siepka S.M., Krentz K.J. (2016). Mouse Tmem135 mutation reveals a mechanism involving mitochondrial dynamics that leads to age-dependent retinal pathologies. eLife.

[72] Serrat R., López-Doménech G., Mirra S., Quevedo M., Garcia-Fernàndez J., Ulloa F., Burgaya F., Soriano E. (2013). The Non-Canonical Wnt/PKC Pathway Regulates Mitochondrial Dynamics through Degradation of the Arm-Like Domain-Containing Protein Alex3. PLoS ONE.

[73] Grossmann D., Berenguer-Escuder C., Bellet M.E., Scheibner D., Bohler J., Massart F., Rapaport D., Skupin A., Fouquier D’Hérouël A., Sharma M. (2019). Mutations in RHOT1 Disrupt Endoplasmic Reticulum-Mitochondria Contact Sites Interfering with Calcium Homeostasis and Mitochondrial Dynamics in Parkinson’s Disease. Antioxid. Redox Signal..

[74] Hartman J.H., Gonzalez-Hunt C., Hall S.M., Ryde I.T., Caldwell K.A., Caldwell G.A., Meyer J.N. (2019). Genetic Defects in Mitochondrial Dynamics in Caenorhabditis elegans Impact Ultraviolet C Radiation- and 6-hydroxydopamine-Induced Neurodegeneration. Int. J. Mol. Sci..

[75] Tsushima K., Bugger H., Wende A.R., Soto J., Jenson G.A., Tor A.R., McGlauflin R., Kenny H.C., Zhang Y., Souvenir R. (2018). Mitochondrial Reactive Oxygen Species in Lipotoxic Hearts Induce Post-Translational Modifications of AKAP121, DRP1, and OPA1 That Promote Mitochondrial Fission. Circ. Res..

[76] Lee S.Y., Kang J.M., Kim D.J., Park S.H., Jeong H.Y., Lee Y.H., Kim Y.G., Yang D.H., Lee S.H. (2017). PGC1α Activators Mitigate Diabetic Tubulopathy by Improving Mitochondrial Dynamics and Quality Control. J. Diabetes Res..

[77] Zhao Y., Tian J., Sui S., Yuan X., Chen H., Qu C., Du Y., Guo L., Du H. (2017). Loss of succinyl-CoA synthase ADP-forming β subunit disrupts mtDNA stability and mitochondrial dynamics in neurons. Sci. Rep..

[78] Park H., He A., Tan M., Johnson J.M., Dean J.M., Pietka T.A., Chen Y., Zhang X., Hsu F.F., Razani B. (2019). Peroxisome-derived lipids regulate adipose thermogenesis by mediating cold-induced mitochondrial fission. J. Clin Investig..

[79] Gal A., Balicza P., Weaver D., Naghdi S., Joseph S.K., Várnai P., Gyuris T., Horváth A., Nagy L., Seifert E.L. (2017). MSTO 1 is a cytoplasmic pro-mitochondrial fusion protein, whose mutation induces myopathy and ataxia in humans. EMBO Mol. Med..

[80] Zeng Y., Pan Q., Wang X., Li D., Lin Y., Man F., Xiao F., Guo L. (2019). Impaired Mitochondrial Fusion and Oxidative Phosphorylation Triggered by High Glucose Is Mediated by Tom22 in Endothelial Cells. Oxidative Med. Cell Longev..

[81] Suomalainen A. (2011). Therapy for mitochondrial disorders: Little proof, high research activity, some promise. Semin. Fetal Neonatal Med..

[82] Rahman S., Clarke C.F., Hirano M. (2012). 176th ENMC International Workshop: Diagnosis and treatment of coenzyme Q (1) (0) deficiency. Neuromuscul Disord..

[83] Montini G., Malaventura C., Salviati L. (2008). Early coenzyme Q10 supplementation in primary coenzyme Q10 deficiency. N. Engl. J. Med..

[84] Rahman S., Hargreaves I., Clayton P., Heales S. (2001). Neonatal presentation of coenzyme Q10 deficiency. J. Pediatr..

[85] Duncan A.J., Bitner-Glindzicz M., Meunier B., Costello H., Hargreaves I.P., Lopez L.C., Hirano M., Quinzii C.M., Sadowski M.I., Hardy J. (2009). A nonsense mutation in COQ9 causes autosomal-recessive neonatal-onset primary coenzyme Q10 deficiency: A potentially treatable form of mitochondrial disease. Am. J. Hum. Genet..

[86] Rahman S., Hanna M.G. (2009). Diagnosis and therapy in neuromuscular disorders: Diagnosis and new treatments in mitochondrial diseases. J. Neurol. Neurosurg. Psychiatry.

[87] Wang Y., Landry A.P., Ding H. (2017). The mitochondrial outer membrane protein mitoNEET is a redox enzyme catalyzing electron transfer from FMNH2 to oxygen or ubiquinone. J. Biol. Chem..

[88] Andreux P.A., Houtkooper R.H., Auwerx J. (2013). Pharmacological approaches to restore mitochondrial function. Nat. Rev. Drug Discov..

[89] Dominy J.E., Puigserver P. (2013). Mitochondrial biogenesis through activation of nuclear signaling proteins. Cold Spring Harb. Perspect. Biol..

[90] Stetler A.R., Leak R.K., Chen J. (2013). The dynamics of the mitochondrial organelle as a potential therapeutic target. J. Cereb. Blood Flow Metab..

[91] Alexander C., Votruba M., Pesch U.E., Thiselton D.L., Mayer S., Moore A., Rodriguez M., Kellner U., Leo-Kottler B., Auburger G. (2000). OPA1, encoding a dynamin-related GTPase, is mutated in autosomal dominant optic atrophy linked to chromosome 3q28. Nat. Genet..

[92] Zuchner S., Mersiyanova I.V., Muglia M., Bissar-Tadmouri N., Rochelle J., Dadali E.L., Zappia M., Nelis E., Patitucci A., Senderek J. (2004). Mutations in the mitochondrial GTPase mitofusin 2 cause Charcot-Marie-Tooth neuropathy type 2A. Nat. Genet..

[93] Waterham H.R., Koster J., van Roermund C.W., Mooyer P.A., Wanders R.J., Leonard J.V. (2007). A lethal defect of mitochondrial and peroxisomal fission. N. Engl. J. Med..

[94] Cassidy-Stone A., Chipuk J.E., Ingerman E., Song C., Yoo C., Kuwana T., Kurth M.J., Shaw J.T., Hinshaw J.E., Green D.R. (2008). Chemical inhibition of the mitochondrial division dynamin reveals its role in Bax/Bak-dependent mitochondrial outer membrane permeabilization. Dev. Cell..

[95] Wang D., Wang J., Bonamy G.M., Meeusen S., Brusch R.G., Turk C., Yang P., Schultz P.G. (2012). A small molecule promotes mitochondrial fusion in mammalian cells. Angew. Chem. Int. Ed. Engl..

[96] Qi X., Qvit N., Su Y.C., Mochly-Rosen D. (2013). A novel Drp1 inhibitor diminishes aberrant mitochondrial fission and neurotoxicity. J. Cell Sci..

[97] Cascella M., Bimonte S., Muzio M.R., Schiavone V., Cuomo A. (2017). The efficacy of epigallocatechin-3-gallate (green tea) in the treatment of Alzheimer’s disease: An overview of pre-clinical studies and translational perspectives in clinical practice. Infect. Agent Cancer.

[98] Perez Ortiz J.M., Swerdlow R.H. (2019). Mitochondrial dysfunction in Alzheimer’s disease: Role in pathogenesis and novel therapeutic opportunities. Br. J. Pharmacol..

[99] Leduc L., Levy E., Bouity-Voubou M., Delvin E. (2010). Fetal programming of atherosclerosis: Possible role of the mitochondria. Eur. J. Obstet. Gynecol. Reprod. Biol..

